# An Essay on Delirium Tremens

**Published:** 1838-10

**Authors:** 


### Art. III.

1. *Commentatio de Delirio Tremente.* Auctore O. C. Höegh-Guldberg, m.d., Medico Nosocomii Fredericiani secundario.—*Hauniæ*, 1836. 8vo. pp. 293.

*An Essay on Delirium Tremens.* By O. C. Höegh-Guldberg, m.d.—*Copenhagen*, 1836.

2. *Prize Dissertation* (*for* 1831) *on Delirium Tremens.* By James Conquest Cross, m.d., of Lexington, Kentucky.—*Albany*, 1832. 8vo. pp. 149.

Delirium Tremens is one of the many diseases which have found investigators and a name only in more recent years. Before the commencement of the present century, no author had made that series of accurate observations on its symptoms and nature which could enable him to distinguish it from those affections with which, in many respects, there exists a very considerable resemblance. Our countryman, Dr. Samuel Burton Pearson, was the first who called attention to its history; and to him we likewise owe the distinctive epithet by which it was originally characterized. In 1801, and subsequently in 1813, he concisely and very accurately describes the symptoms of this disease under the title of Brain Fever *following intoxication.* On referring to his papers, then published, it is gratifying to see that the treatment he recommended and adopted is almost the same as that which, in the present day, is esteemed to be the most judicious.

The work of Dr. Höegh-Guldberg is essentially a practical work, and is especially valuable from the extensive opportunities he has enjoyed of witnessing cases of this disease. The deductions drawn from the observations he has made, both as regards its nature and treatment, are well worthy of attention.

Dr. Cross's book is one quite of a different description. His opportunities of testing the correctness of his own opinions, and those of others, do not seem to have been extensive; but he has furnished us with a very excellent and comprehensive literary history of the disease: he has placed fully before us the different views which have been entertained by those authors who have written on the subject; and, from having canvassed judiciously their relative merits, has thus presented us with a very copious digest of what is at present known about delirium tremens.

The number of names by which this disease has been designated is evidence sufficient of the difficulty there exists in referring its symptoms to some certain type. The following catalogue, which we extract from Dr. Höegh-Guldberg's work, contains only a part of the synonymy of delirium tremens. Even this bare nomenclature is not devoid of interest. It will be observed that the fancied seat of the disease or its remote cause has furnished many authors with names for it; while others have derived these from the more prominent symptoms.

"Phrenesia potatorum, (Albers); delirium afebrile tremens, (Graff); oenomania, (Rayer); erethismus cerebri abdominalis, (Toepken); delirium vigilans, (Hayward); delirium ebrietatis potatorum, (Hufeland); encephalitis tremefaciens, (Frank); mania a potu, (Nancrede); mania a temulentia, (Klapp); mania potatorum, (Barkhausen); ebriositas sive hallucinatio ebriosorum, (Clarus); dipsomania, (Hufeland); delirium nervosum, (Ryan); erethismus ebriositatis, encephalopathia nervosa, (Leveille); polydipsia ebriosa, (Henke); ecstasis nervosa, (Clarus); delirium nervosum s. traumaticum, (Dupuytren); delirium cum tremore, (Elliotson); brain fever following intoxication, (Pearson); irritative fever of drunkenness, (Tripler); tremulence and delirium from intemperance, (medicus quidam americanus); encephalopathie crapuleuse, (medicus quidam gallicus); denique delirium tremens, (Sutton.)" (p. 6.)

Dr. Cross has entered into some discussion on the relative value of these and other names; but into this we cannot follow him. We entirely agree, however, in the propriety of adopting the term most generally received, viz. *delirium tremens;* although we admit, with him, that it is "highly objectionable." In this, as in other cases, when we consider how impossible it is accurately to comprehend in a name the series of symptoms which constitute to the senses of a physician the disease itself, we had at all times better be contented with an empirical epithet which has from universal consent become established, than involve the subject with fresh difficulties by seeking for another name, which, though it may perchance be somewhat preferable, yet can rarely, if ever, from the diversified nature of disease, be entirely free from objection.

As with the name, there is the greatest difficulty in arriving at any thing like a true and concise definition of this disease. The attempt is, however, hazarded by Dr. Höegh-Guldberg. "Delirium tremens," he says, "is a disease which, in the first place, shows itself by aberrations of the functions of the cerebrum and nerves generally as evinced in *pervigilia*, delirium, hallucinations on some peculiar subject, together with (often) a trembling of the limbs: it is almost always associated with fever, (in which respect, he adds, his observations differ somewhat from those of other writers who have described it;) is very prone to occasion collapse; and is never recovered from but with a critical sleep: it has its principal and almost only cause in a daily abuse of spirits."

Though this definition may in the main be true, yet in many cases it fails of its purport; and there is no disease in which more mischief could arise by tying down one's opinion strictly to the symptoms detailed in a definition. That this disease may have other origin than the habitual abuse of ardent spirits, no one who has enjoyed extensive opportunities of witnessing cases can doubt, any more than that occasionally there is an attendant sthenic fever, now a *tremor artuum*, and now none; and that cases not unfrequently occur where, with all the other worst symptoms, there is no delirium, but only an excitability and restlessness of mind. In fact, a true knowledge of this most opprobrious malady can only be arrived at by studying its symptoms under many different circumstances. We have ourselves witnessed enough of this disease to enable us to conclude that there is no one symptom which is constant or essentially characteristic of its presence. A case came lately within our observation, in the person of a female, whose station in life and ample means should have inspired her with more creditable habits, where the only departures from health were a general irritability of manner and a morbid impression that her legs had become black and were ready to separate from her body: the constant examination of her legs, and her anxiety to draw attention to that supposed condition, were the only external evidence of her labouring under delirium tremens. In another case, which came under our notice, a gentleman of considerable literary attainments, addicted to irregular and occasional excess only, rose suddenly from the head of his own table, where he had been partaking too largely of wine (the only drink of an intoxicating nature he indulged in) in a temporary but very violent phrensy, which quickly subsided into sobs and fears lest his servants should betray his infirmity. In both these instances a critical sleep was the restorer to a healthful state.

When speaking of the etiology of this disease, we think our author a little too strong in his expression when he asserts "that all writers agree in saying that this disease is produced by the abuse of ardent spirits, and that in this abuse both the remote and predisposing causes are to be sought; and, that this assertion is true, daily observation, as well as the confessions of the sick themselves, have confirmed." (p. 9.) That this is by far the most frequent, nay, nearly the only cause of the disease, there can be no doubt; but it most assuredly can by no means be asserted to be exclusively so. Dr. Höegh-Guldberg, in another part of his work, somewhat modifies this statement, by allowing that there are other substances which may produce symptoms having a resemblance, more or less marked, to delirium tremens: " Among the substances which may give rise to symptoms bearing greater or less similitude to this disease may be enumerated various narcotics, as belladonna, stramonium, and especially opium." (p. 18.) There can, however, be no doubt, as above stated, that, although amongst us the chief and almost only cause of this disease is the abuse of ardent spirits, yet that most certainly delirium tremens may have its origin in several other causes; and that in these cases it not only bears " a greater or less similitude" to, but is essentially the disease itself. One reason why it more rarely has its origin in these other substances than in ardent spirits may be the fact of the very general abuse of the latter; while the former are but rarely used, excepting for medical purposes. In contradiction to the statement of Dr. Höegh-Guldberg, we offer the few following instances: Dr. Armstrong gives us the case of a female, who had long been in the habit of taking opium to a considerable extent. On lessening her daily quantity, she became a victim to this disease. Dr. Coates also says he has seen strongly characterized cases, in which it was produced by the intermission of the use of opium. Höring refers to the case of an old man, in whom he witnessed this disease after the long abuse in strong coffee.* Dr. Sutton saw it in a lady, after an habitual indulgence in the use of red lavender.† Dr. Barkhausen, who affirms that he has seen this disease, in a mild form, follow the use of strong beer, doubts if it ever arise from wine. This appears a strange reservation on the part of this well-informed physician. The case of the gentleman to which we have referred is, however, proof to the contrary. It would, no doubt, be difficult to prove, from a great number of cases, that wine is a remote cause of the disease, from its very rarely happening that those who indulge very deeply in vinous drinks altogether refrain from spirit; but there is every analogy to induce us to

* Kleinert's Repertorium. Oct. 1833.

† In this instance, however, the disease may be attributed solely to the alcohol with which this tincture is made.

conclude that an abuse of wine solely may produce it. Dr. Cross justly remarks "that this disease may proceed from wounds, fractures, &c.: the *delirium traumaticum* of M. Dupuytren does not appear essentially unlike *delirium tremens* in its characteristic features;" and Dr. Gregory, in his Practice of Physic, refers to a case he once saw, in which "a distinct affection of the brain, by metastasis of acute rheumatism, assumed the very remarkable character of *delirium tremens*." These cases, and many more might be quoted, sufficiently show that our author has been too restrictive in limiting the origin of this disease to the abuse of ardent spirits only.

Dr. Höegh-Guldberg, when discussing the remote causes, has omitted the by no means unimportant questions, whether a protracted course of immoderate drinking is always essential to the production of the disease; and whether, as many suppose, there must be a suspension in the use of ardent spirits immediately preceding the attack. With regard to the former of these questions, the general impression seems to be that a long course of dissipation is necessary to cause an attack; and, although for the most part this certainly is the case, yet there can be doubt it does not always hold good. In persons of irritable temperament, and liable to præcordial anxiety, a single excess is sufficient to produce it; but then the disease, though it may be as acute at the onset, is not of the same continuance or fatal tendency as when preceded by a debilitating course of dissipation. It also occasionally occurs in those individuals who, never drinking to that excess which produces drunkenness, are frequently in the habit of allaying a constant thirstiness by stimulating drinks. Dr. Barkhausen says that he has seen it "in those who have seldom, or perhaps never, become intoxicated." We think we have seen the same; and therefore conclude that this disease is not wholly to be attributed to the quantity of spirits drunk at one time, or to the period in which the habit has been indulged in: constitution and other circumstances must also be taken into consideration.

"The natural strength and irritability of the constitution," says Dr. Cross, "will always exercise great influence in modifying the result. Thus, when there is but little constitutional strength and considerable nervous irritability, ardent spirits will produce much higher excitement than where the circumstances of the individual are directly the reverse. If this be true, the legitimate conclusion is that delirium tremens will be developed with the least promptitude in the constitution which most reluctantly comes under the influence of unnatural or spirituous stimulation." (p. 20.)

With regard to the second question, as to whether it is necessary to the production of the disease that the use of ardent spirits should be suspended, we can have no hesitation in stating, from personal observation, that it is not requisite; though undoubtedly the attack is most usually preceded by a state of depression resulting from the temporary discontinuance of the wonted stimulus.

One of the most valuable parts of Dr. Höegh-Guldberg's essay is the deductions from observations made upon 173 cases of this disease, which came under his own observation. From these we are enabled to see the relative influence of age, sex, and season. Dr. Bang, Lind, and Rayer appear to think that the disease occurs more frequently between the age of thirty and fifty; whereas, our author limits its greatest frequency to the period between forty and fifty. He witnessed it but once in a young man of twenty-two years of age, and but once in an old man of seventy. With regard to its frequency in women, Rayer observed it in seven women out of 176 cases. Bang states that, in 456 cases which he treated, only ten were females; and from the tables of our author we find but one case out of 173 occurring in a female. Krüger-Hansen in sixteen cases, and the chief physician of the hospital Christiana in eleven cases, met with no instance in a female. These statements differ very much from the accounts given in this country; for we find both Dr. Ryan and Dr. Roots state that the sexes are equally obnoxious to its influence. This discrepancy can only be explained by supposing that the indulging in intoxicating drinks amongst females is carried to a greater extent in this country than in the north of Europe.

With regard to the influence of season, the month of May appears to be the time when attacks are most frequent. Dr. Höegh-Guldberg observed in this month twice as many cases as in any other month. This does not altogether accord with the observations of Bang, who states that in June and July cases are as frequent as in May, but that the mass of cases occur during these months. It appears evident from the above, as well as from the following facts, that atmospheric phenomena exert a considerable influence over this disease. Between the years 1826 and 1829, of nine thousand sick in the hospital at Frederickstadt, 456 were cases of delirium tremens; while, in the period from 1830 to 1832, amongst seven thousand patients in the same hospital, only 173 were cases of this disease.

From a synoptical view of the 173 cases above mentioned, given at the end of the work, we obtain some other statistical results of importance. Thus we learn that, of this number, 149 were cured, (twenty-two of these not having undergone any curative means but quiet,) and twenty-two died; that the critical sleep occurred in the greater number on the fourth day; that the period of life most obnoxious to the attack was from the fortieth to the fiftieth years; that it occurred mostly in street porters, literary men, and tavern keepers; that, in 159 cases, it was, without doubt, brought on by the abuse of spirits, and most probably so in the remaining fourteen; that, of those who took opium, eighty-three were cured, and thirteen died; that, of those who were bled, eleven recovered, and seven died; and that a deposit of lymph between the membranes of the brain was the most usual appearance after death. In another part of the volume, a very full account is given of the appearances on dissection, occupying a space of thirty pages: but we do not see that the observations essentially differ from what had previously been noted, nor do they tend to throw any new light upon the pathology of the disease.

In the history of the symptoms and general character of delirium tremens, Dr. Höegh-Guldberg very properly draws a distinction between those cases which occur in otherwise healthy persons, the disease having its origin solely in the abuse of spirits, and those cases where it is complicated with other lesions, either internal or external. The sad condition to which the state of health is reduced in the confirmed drunkard is ably summed up by Dr. Höegh-Guldberg, in the history of the precursory signs.

"An almost constant precursor of this disease is great disturbance of the digestive organs; the long indulging in the abuse of ardent spirits has destroyed the appetite for food; a mucous and nauseous taste is felt in the mouth; the food which is taken in is often rejected, especially in the morning; oppression and tension of the epigastrium are felt, with flatulence and general anxiety; which symptoms, though they may be for a time allayed by taking spirits or bitters, progressively increase. At length the temporary relief afforded by these means is worn out, or else a dislike is taken to them. Then the gastric evils become intolerable; vomiting supervenes, and mucous matter, sometimes tinged with bile, and more rarely with blood, is evacuated: this state is often accompanied by a torminous diarrhœa. Occasionally the patient is attacked by epileptic fits; and some medical men have so constantly observed these to take place, that they have been convinced that, within twenty-four hours, an attack of the disease would commence: sometimes an epistaxis happens, so considerable as, if neglected, to continue for many days; sleep begins to leave the patient's eyelids; his mind undergoes a complete change; his ordinary business is less pleasing to him; he becomes morose, irritable, and obstinate, impatient of almost every contradiction, especially from those with whom he is familiar; while the anxiety which he suffers he desires and uses every endeavour, but in vain, to conceal from the observation of others." (p. 27.)

Although this account of the dilapidated health caused by a long series of dissipation is very correct, yet we are convinced that such a condition cannot be called precursory to an attack of delirium tremens; for, however susceptible of this disease a person reduced to such a state may be, yet it is by no means a consequence that an attack should follow; and, on the other hand, many who come within the influence of the disease have not been previously sickened down to any such lamentable extremity. We by no means desire, however, to take from the force of the above statement that such a condition is in every way likely to predispose to the disease. The remark that epileptic fits are constantly and immediately followed by an attack of delirium tremens, is no doubt founded in error; for we have not unfrequently seen these convulsions produced in the drunkard, and that, too, in the most aggravated and distressing form, without the severer disease supervening.

"In the first stage, the precursors are mingled with the disease itself. Though still of sound mind, yet the patient complains bitterly of a troubled sleep: scarcely are his eyes closed when a whole host of phantasies obtrude upon him: he seems to hear various sounds, the causes of which, rising up with fright, he at first vainly endeavours to discover: at one time he thinks them to be in his own ear, and then that they proceed from a distant spot; sometimes he attributes them to the agency of demons, which seem to blow air into his ears; and yet, singular to say, he is sensible of these errors of his senses. If he be roused from sleep, in the first moment he knows not where he is, or to whom he speaks: he complains of vertigo, a feeling of weight in the head, and loss of memory; and an inconstancy and unsteadiness are evident in his thoughts and words, and in all the voluntary motions. In this early stage of the disease, the patient accommodates himself, to a certain extent, to the will of others: sometimes he makes replies so apt to the questions put to him, that the existence of the disease might be mistaken, were it not for a certain inconstant expression of countenance, a silly distraction of mind, or perhaps from a sudden and unexpected motion, by which he endeavours to seize a thread or insect, the phantasms of his own imagination, which he believes to be affixed to the floor or bedclothes. If he be asked what he sees, or what he endeavours to lay hold of, he will tell you; adding, that he was in error; and then, for some little time, his replies are sufficiently apposite and coherent. At the request of his physician he sometimes goes to his bed, and for a short period composes himself for sleep; then he rises, and seeks for one thing or another among the bedclothes; or he replies to something he imagines to be addressed to him." (p. 29.)

In this first stage the patient may be permitted, with proper care, to walk about; and it may happen that the symptoms do not further increase: this, however, is seldom the case. Should he not be refreshed by sleep, the disease will certainly increase, especially towards night, when hallucinations of the senses, delirium, and trembling of the limbs supervene; in fact, all the essential symptoms of the confirmed disease. This is the stage which our author terms the *incrementum morbi.*

"The restless hilarity of the patient now increases: he talks much, and to questions often gives sufficiently apt and even witty replies; then, after a little time, forgetful of the tenor of his discourse, he answers questions made to others, or does not understand those addressed to himself. It is not unfrequently a source of much fear to him that his medical assistant may not effect that which he desires. He is irascible, angry, or even furious; yet, for the most part, if not strongly contradicted, he may still be controlled. During his hallucinations, he almost always thinks himself to be engaged in his habitual pursuits. The postboy is driving his horses—he cheers them, he beats them; the servant appears to hear the voice of accustomed command, and, mistaking some other individual for his master, offers him his services; the watchman calls the hours; the sailor works his ship; the porter endeavours to move from its place the table, bed, &c. This restlessness continues to increase with the delirium, until, exhausted by exertion, and covered with perspiration, the poor victim subsides into a temporary quiet. It more rarely occurs, though many such cases are not wanting, that the mind becomes overwhelmed with melancholy, with religious forebodings, &c. These people do nothing, or else seem unable to do what they desire; or they wander up and down the room without purpose, or seek to hide themselves from importunate creditors or the officers of public justice. If at this time they be suffering from any local pain, all anxiety and attention is referred to the spot, and it has sometimes been observed that they attach to themselves any pain they may at the time hear complained of. Barkhausen gives the case of a man who supposed himself pregnant, from hearing his wife's complaints during labour. The patient's mind is taken up occasionally with the strong desire of ardent spirits: his joy is raised because he thinks it is before him; he asks for it with eagerness, and the cup offered to him is not refused; but his taste is often lost; whatever be in the cup he drinks it, believing it to be spirits; at another time, however, he is not thus deceived." (p. 32.)

Very frequently the sense of sight is greatly depraved, the hallucinations being extremely diversified, yet for the most part more or less referable to certain forms. The cause of these Dr. Höegh-Guldberg thinks to be congestion of the capillary vessels in the transparent parts of the eye. The sense of hearing is also particularly affected: imaginary conversations are heard, in which the patient joins; spirits laugh, and the patient emulates their mirth; tempests roar, dogs howl, &c. &c. The only sense which is unaffected is that of touch.

In defining the commencement of the third stage, Dr. Höegh-Guldberg follows Göden, who says this stage commences when the patient loses *all* consciousness of his past and present condition.

"He knows not where he is, or to whom he speaks; altogether inattentive to external objects, he follows out his own ideas; the delirium is constant and violent; the expression of the countenance is fierce and immoveable; the eyes are closed, the lids being frequently covered by an acrid secretion; the conjunctiva both of the ball and lids is inflamed; the pupil, which was before dilated, becomes contracted, and seems scarcely sensible of light. To one unaccustomed to witness this disease, the strength would appear exhausted; but, on the advent of a paroxysm, it is so considerable as to require much power to control it. Sometimes the tremors of the limbs pass into a spasmodic rigor of the whole body, and the patient is suddenly prostrated on the ground, without the power of rising; but he soon recovers, and resumes his previous state of restless occupation. This stage may last from one to six days." (p. 38.)

The fourth stage is computed from that time when frequent remissions, lassitude, and prostration of strength supervene, and can only be said to have fairly set in when sleep overtakes the exhausted patient: sleep is the only natural and salutary crisis in this disease. It must be particularly noticed that this condition is not sudden in coming on: for some time manifestations of a sleepiness are present, together with an increasing tranquillity both of mind and body; but yet some space elapses before the patient is perfectly free from hallucinations: at length, however, he gradually passes into a state of repose, which ends in a true sleep, the prelude of a speedy, sometimes of an immediate recovery. If, however, in place of this critical sleep, the restlessness continue, or if the patient be roused up with tremors and convulsions, we are justified in expecting a speedily fatal termination.

The critical sleep is a subject worthy of much attention: it frequently continues for twenty-two or twenty-four hours, and in some cases even for thirty-six and thirty-eight; only being disturbed by natural causes. A sleep of only six or eight hours is, however, frequently critical, being followed by recovery.

Whether delirium tremens is to be considered as of a febrile nature or not, has been the cause of much dispute. The latter opinion has been maintained by Göden, Blake, Dupuytren, and others; while Coates, Barkhausen, and Armstrong, as also Dr. Höegh-Guldberg, hold to the former. He says he has never known the pulse less than eighty or ninety, more frequently upwards of one hundred; but, in the generality of cases, 110 or 120, and not unfrequently 140.

The following are some of Dr. H.-G.'s observations on the symptom which gives its name to the disease:

"*Tremor artuum* generally happens amongst all drunkards, even before they are attacked by this disease; especially if a life of habitual dissipation has undermined the constitution. That it sometimes is not present cannot be denied, but the exceptions are only met with in young or robust men, who, from some accidental cause or another, have become affected with this disease before the constitution has been destroyed by habitual drunkenness. For the most part, the hands are the parts which are affected with the greatest tremor; though not infrequently the arms, and occasionally even the whole body, suffers. In some cases the tremor becomes so considerable that the patient is unable to lay hold of small objects; nor can he raise, without spilling the contents, a cup to his mouth. In the more aggravated cases, it prevents his walking steadily unless supported by others; if he rise from his seat, he totters, and the eagerness with which he endeavours to restore his lost equilibrium, and the strenuous efforts made to grasp some object for support, only increase the tremor. As the disease advances, the organs of speech become affected; the tongue being, contrary to the will, thrust out, or retracted or fixed between the teeth." (p. 52.)

Under the head of *Delirium tremens chronicum*, Dr. H.-G. well describes that chronic condition of imbecility of mind and prostration of body to which the habitual drunkard becomes liable. In the following chapters, which treat of the Prognosis and the Diagnosis, we also find much valuable matter; but we pass over these to notice the more important subject of the Treatment. Dr. H.-G. is of opinion that much time has been unnecessarily consumed in fixing on the indications of treatment. He thinks it sufficient if it be borne in mind that the first object to be obtained is the critical sleep; "for, whether the physician select the antifebrile or antiphlogistic mode of cure, or the exciting or sedative, he does nothing else, nor is anything else to be done, than prepare the way for sleep, by lulling the disturbed state of the vascular and nervous systems." It sometimes happens that recovery from this disease takes place spontaneously; but the result of such rare cases is only to impress upon the mind the advantage of treatment. Dr. H.-G. says that he has seen the best effects from a cool regimen, spare diet, internal refrigerants, and the application of cold lotions or cold affusions to the head. In the sthenic variety he prefers the use of the lancet, observing that in this form, which for the most part occurs in robust young men, and after excesses newly committed, local congestions are to be especially guarded against.

He is persuaded that this form of delirium tremens is, in fact, a species of arachnitis; and that the congestion in the brain, attended, as it is in young and robust men, with a full, strong, and tense pulse, is of such pressing moment as to indicate immediate bloodletting. This practice, he adds, can however be only safely pursued in those whose health has not been weakened by the occurrence of previous attacks or a debilitating course of dissipation, or in whom the disease has existed but for a short period; for, in the latter stages of the disease, when there is always great debility and a tendency to sudden collapse, it must be instituted only with much caution, or altogether avoided.

When venesection is proper, he recommends great circumspection as necessary in regard to the quantity to be abstracted; observing, that he had ever found twelve ounces sufficient, and that he never repeated the operation unless there remained unaltered some marked sthenic character of the disease. He has frequently observed the symptoms remit so quickly after this mode of treatment, that the critical sleep has supervened within an hour. After passing in review the names and opinions of many of those who have either maintained the efficacy or have entirely condemned the use of the lancet in this malady, he sums up by saying that the middle course is to be pursued, and that these latter are in as great error as those that commend its promiscuous use. Local bloodlettings, either by leeches or by cupping, he says, may be employed with less reserve than general bleeding. They may be resorted to at the commencement of the disease, if such indications as have just been referred to, when speaking of general bleeding, be present; as likewise in the later stages of the disease, should symptoms of congestion in one or other of the organs of the principal cavities manifest themselves, and when venesection is precluded. The use of local bloodletting is particularly indicated if heat or congestion of the head, attended by vertigo, singing in the ears, and redness of the eyes, continue.

Dr. H.-G. is particularly partial to the employment of cold applications and affusion, the use of which is indicated, in young and strong persons, by the presence of urgent and continued congestion of the brain, with redness and heat of face. He says there does not appear to be any better remedy for controlling the continually rising hallucinations and phantasies, which unbidden crowd on those afflicted with delirium tremens, in the first stage of the disease; but he entirely precludes their employment in the subsequent stages, and says that, when used at such times, he has seen universaltremors occur, occasionally so considerable as to similate convulsive motions. Lind, Barkhausen, Burrow, Armstrong,

Albers, and others, who have found the use of cold affusion serviceable, caution us against its employment if any profuse perspiration be present, recommending in such cases the employment of the tepid affusion. Dr. H.-G. has, however, no reserve of this kind, but adds further, that such cases very rarely occur; for, in the stage of the disease in which cold affusion is serviceable, or is indeed only practicable, the head is dry and hot, and rarely if ever suffused with perspiration. He has observed that the mind is somewhat steadied, or even recovered, after every affusion; and therefore recommends that this remedy should be repeated frequently, (every two hours,) and so continued until the head becomes perfectly cooled.

Following Horn and Richter, Dr. H.-G. has tried cold affusion to the head while the patient has been immersed in the tepid bath, and not without success; but this practice is almost precluded by the difficulty which attends the carrying it into effect; for those affected by delirium tremens almost universally resist the tepid bath. Richter, however, affirms that its efficacy is so great that he has frequently found the use of the bath, together with the employment of nauseating medicines, effect a recovery within a few hours. Cold applications to the head may be applied after the cold affusion, though it rarely happens that the patient is sufficiently tranquil to render this course practicable: should it be so, however, they are very useful. Their efficacy may be increased by their being mixed with slight derivatives, as mustard: the stronger rubefacients and vesicatories are most useful in the asthenic form of the disease, and should not be used in the sthenic, unless to overcome some dullness of the mental faculties, which might otherwise become permanent.

Of the use of laxatives, Dr. H.-G. has had but little experience: in those cases, however, in which they were employed, the sulphate of magnesia was resorted to. He makes the observation that delirium tremens frequently occurs in those labouring under diarrhœa, and that such condition of the system does not alleviate the delirium.

In about half the cases that came under his care he administered the nitrate of potass; not, he says, because it is endowed with any specific power of controlling this disease, and in which other cooling medicines are deficient, but that it is a febrifuge which he has been accustomed to use unless contraindicated. He believes it to be only useful at the commencement of the disease, and quite inefficacious if the disease have reached its acme.

Of the supertartrate of potass and sal ammonia the same may be said.

The frequent complication of delirium tremens, during one year, with typhus, induced him to try the effect of acids diluted by water or spirit; a remedy much praised by Albers and Barkhausen. He found it successful at the commencement as well as in the latter stages of the disease, and recommends it as being greatly serviceable when delirium is superadded to wandering and restlessness of mind, and likewise when the thirst is more intense, and the sweatings greater, and the tremor more violent than is usual in the sthenic variety of the disease. When symptoms such as these manifest themselves, as is not unfrequently the case, five or six drops of Haller's elixir* he often found restore the tranquillity,

* ℞. Acid. Sulph. dil. ʒj.; Aquæ Ceras. nig. ℥iv.; Syr. Rub. Id. ℥j. M. Radius Heilformeln, p. 14. Leipzig, 1836.—ED.

and in some measure subdue the *tremor artuum.* He recommends, in order to satisfy the morbid fancies of the patient, that medicines should be administered in a cup similar to that from which he has been in the habit of drinking his intoxicating draughts.

On examinations after death, the frequent appearance of inflammation and the formation of lymph on the membranes of the brain, together with the recommendations of Lind, Armstrong, Gunther, and others, induced him to try the effect of calomel in "accustomed doses, and according to accustomed rules," (two grains every alternate hour.) This he did, however, in those cases only in which the delirium tremens was complicated with inflammations of the chest, and in which cases he found it very useful.

Dr. H.-G. canvasses at some length that mode of treatment by emetics so much recommended by Stoll, Göden, the American physicians, and others. The doses of tartar emetic which it has been the custom of some of these to administer appear, indeed, surprising to the British practitioner; for we find Stoll giving from four to seven grains, and Sperce (Spence?) administered the enormous doses of thirty grains, which he repeated every hour, and even every half hour. This last writer assures us that the effects of these doses were an immediate lassitude, a slight vomiting and diarrhœa, tranquillity of body and mind, and then the critical sleep. Dr. H.-G. never prescribed it to an extent anything like this.

It has been asserted that, after the injudicious and continued use of tartar emetic, vesicles are formed in the cavity of the mouth: these Dr. H.-G. has never seen, although, during the year preceding that in which he wrote, large numbers of cases have been treated by this medicine; but he has often seen on the tongue foul and deep ulcers, caused most probably by its being bitten during the epileptic fits which so often precede attacks of delirium tremens. He also says he has never noticed, after the use of tartar emetic, though he has employed it in gastric and bilious fevers and in affections of the chest, those inflammations of the membranes of the bowels which have been observed by Orfila, Dupuytren, and Trousseau; yet that, having no doubt of their occurrence, he was induced to be very cautious in the use of this medicine. After general or local bloodletting, he administered half a grain in solution: if this did not produce nausea, he increased it to a grain, and then to a grain and a half: and this treatment he continued for two or three days, if the excitement of the nervous and vascular systems did not yield. The greatest amount of tartar emetic he ever administered during the whole course of the disease was forty-three grains. The usual effects which he observed to follow its use were vomiting, diarrhœa, and depression for two or three days. Take it altogether, he does not approve of it, and thinks it unworthy of the praises which have been bestowed upon it. He also tried ipecacuanha, and ipecacuanha combined with tartar emetic; but did not find that their use was attended by any very marked beneficial results. In one case, however, after employing bloodletting, nauseating and other medicines, for some days without avail, he administered the tartar emetic in conjunction with the infusion of digitalis, and they were responded to beyond all hope. The patient, before ungovernable, became tractable, sought his bed, and fell into the critical sleep. The tartar emetic, combined with nervous medicines, as valerian, &c., he found useful in the later stages of the disease.

In the asthenic, that is, the more common form of the disease, opium is, with Dr. H.-G., as with us, the main remedy. After canvassing the various opinions and practice of other authors respecting its employment, he informs us that his own observations have led him to conclude that, although it is a safe and most valuable remedy, it should be used with more caution than is wont to be the case. Though he very commonly administered it (in about half the cases treated), he yet gave it in no one instance to a greater extent than forty-eight grains during the whole course of the disease. The more usual quantity, and that which he preferred, varied from two to ten grains. He says there is no particular preparation required before administering opium. The white mucous tongue, the flatulence, and the oppression at the cardia, to which drunkards are liable, and the morning vomitings under which they labour, often for many months, do not contraindicate its use. Should there, however, exist any severe gastric affection, or any congestion, either general or local, means must first be resorted to for their removal: but such condition belongs rather to the first stage of the disease, a period when the employment of opium is improper. He states the fit time for its exhibition to be the beginning of the third stage, which is characterized by the loss of present and former consciousness. If, on using it, symptoms of apoplexy supervene, as coma, stupor, difficult or stertorous breathing, &c., it must, of course, be discontinued.

He makes some few observations upon the relative value of many other remedies, as valerian, camphor, musk, &c.; but, as these are but of very secondary importance, we pass them over without further comment; as likewise the observations upon the treatment of the complicated and chronic forms of this disease.

Before concluding, we must again turn for a moment to Dr. Cross's essay. We have already characterized it as being an excellent digest of the facts, views, and opinions of others; and we regard the conclusions he has deduced from them as, for the most part, fair and unexceptionable. But the work is throughout disfigured by a very dogmatic tone, and displays frequent passages of the absurdest grandiloquence and bad taste. We quote a few specimens, taken at random, from two or three adjoining pages:

"In the language of Demosthenes, the cause of medical science might have been saved an Iliad of misfortunes, had physicians been more temperate in the commendation and denunciation of this Herculean remedy. Like woman, however, they are rarely mindful of the advice given by Phœbus to Phaeton: *in medio tutissimus ibis.*" (p. 81.) . . . . "If, therefore, in the succeeding pages, independence should characterize the expression of our practical opinions, the reader should not ascribe it to an overweening self-confidence, but to the irresistible conviction of their truth. Should the author sacrifice the sensibilities of the heart, and disregard the dictates of the head, in order to float buoyantly and gracefully along upon the *aura popularis?*" &c. &c. (p. 82.)

"By the pulse in this variety it is unsafe to be governed: it will not be found as faithful a guide as Great Heart in Pilgrim's Progress. On the contrary, it is in this variety too frequently a perfect *ignis fatuus.*" (p. 87.)

